# Type 3 Fimbriae Encoded on Plasmids Are Expressed from a Unique Promoter without Affecting Host Motility, Facilitating an Exceptional Phenotype That Enhances Conjugal Plasmid Transfer

**DOI:** 10.1371/journal.pone.0162390

**Published:** 2016-09-14

**Authors:** Jonas Stenløkke Madsen, Leise Riber, Witold Kot, Alrun Basfeld, Mette Burmølle, Lars Hestbjerg Hansen, Søren Johannes Sørensen

**Affiliations:** 1 Department of Biology, University of Copenhagen, Copenhagen, Denmark; 2 Department of Environmental Sciences, Aarhus University, Aarhus, Denmark; 3 Max Planck Research Group Chromosome Organization and Dynamics, Max Planck Institute of Biochemistry, Martinsried, Germany; Universite Clermont Auvergne, FRANCE

## Abstract

Horizontal gene transfer (HGT), the transmission of genetic material to a recipient that is not the progeny of the donor, is fundamental in bacterial evolution. HGT is often mediated by mobile genetic elements such as conjugative plasmids, which may be in conflict with the chromosomal elements of the genome because they are independent replicons that may petition their own evolutionary strategy. Here we study differences between type 3 fimbriae encoded on wild type plasmids and in chromosomes. Using known and newly characterized plasmids we show that the expression of type 3 fimbriae encoded on plasmids is systematically different, as MrkH, a c-di-GMP dependent transcriptional activator is not needed for strong expression of the fimbriae. MrkH is required for expression of type 3 fimbriae of the *Klebsiella pneumoniae* chromosome, wherefrom the fimbriae operon (*mrkABCDF*) of plasmids is believed to have originated. We find that *mrkABCDF*s of plasmids are highly expressed via a unique promoter that differs from the original *Klebsiella* promoter resulting in fundamental behavioral consequences. Plasmid associated *mrkABCDF*s did not influence the swimming behavior of the host, that hereby acquired an exceptional phenotype being able to both actively swim (planktonic behavior) and express biofilm associated fimbriae (sessile behavior). We show that this exceptional phenotype enhances the conjugal transfer of the plasmid.

## Introduction

Two distinct general behavioral states are currently recognized in most bacteria: a planktonic state and a sessile state, with the latter initiating biofilm formation. Biofilms are densely packed bacterial communities, encased in a self-produced polymeric matrix, and typically associated with a surface or interface. Bacteria in the planktonic state are normally not associated with other cells or surfaces, and they are actively motile, if capable [1]. Central to the regulatory control of the shift between the sessile and the planktonic state, in members of *Enterobacteriacae* and many other *Bacteria*, is a system that directs and responds to the secondary messenger cyclic-di-GMP (3’,5’-cyclic diguanylic acid). Lowered levels of c-di-GMP stimulate a planktonic state, while high levels induce the biofilm phenotype [2].

C-di-GMP is synthetized via diguanylate-cyclases (DGCs) containing GGDEF domains that catalyze the conversion of 2 × GTP into c-di-GMP. The removal of c-di-GMP is mediated by phosphodiesterases (PDE) that contain EAL or HD-GYP domains. EAL and HD-GYP PDEs hydrolyze c-di-GMP into pGpG and 2 × GMP, respectively. The intra-cellular level of c-di-GMP increases or decreases, via DGC and PDE activity, respectively, as a response to the physiological state of the cell and to external clues in the immediate environment [3], such as temperature [4] and electron acceptor availability [5], but also to signals such as quorum sensing autoinducers produced by bacteria [6–8]. Proteins bind c-di-GMP specifically in order to facilitate such responses and therefore typically mediate a shift towards the planktonic or the sessile cell state, in accordance with the intracellular levels of c-di-GMP [2].

A key factor in sessile biofilm formation and virulence of *Klebsiella pneumoniae* is the expression of type 3 fimbriae, which are thin protein appendages that protrude from the cell exterior and mediate adherence to both biotic and abiotic surfaces [9, 10]. Type 3 fimbriae are recognized as a virulence factor specifically associated with urinary tract infections (UTIs), where the ability to bind abiotic surfaces can extend complications in UTIs associated with foreign bodies such as catheters [11]. Type 3 fimbriae are chromosomally encoded by the *mrkABCDF* operon, which is expressed via the P_*mrkA*[Kp]_ promoter located 204 bp upstream (transcriptional start site) of the *mrkA* gene [10]. Type 3 fimbriae are typical chaperon-usher type fimbriae: MrkA is the major fimbrial subunit, MrkB a chaperon protein [12, 13], MrkC an usher translocase, MrkD the fimbrial tip adhesin [14, 15], and MrkF has a putative assembly/stability function [16]. The regulatory genes *mrkHI* and *mrkJ* are located immediately downstream, but transcribed convergently, of the *mrkABCDF* operon [17]. MrkH is a c-di-GMP-dependent transcriptional activator of P_*mrkA*[Kp]_, controlling the expression of the *mrkABCDF* operon. MrkH binds the secondary messenger c-di-GMP via a PilZ domain, which prompts MrkH to bind upstream of P_*mrkA*[Kp]_, hereby greatly enhancing the activity of transcription of this otherwise very weak promoter, and boosts the expression of type 3 fimbriae [17]. MrkI is a transcriptional regulator that also positively activates transcription of both *mrkHI* and *mrkABCDF*. The molecular function of MrkI is less well resolved [18]. MrkJ is a PDE that catalyzes degradation of c-di-GMP via its EAL domain. MrkJ therefore negatively regulates type 3 fimbriae formation indirectly by reducing the local c-di-GMP pool [19].

The *mrkABCDF* operon is not only found on the chromosome of *K*. *pneumoniae* but also encoded on the chromosome of various other members of *Enterobacteriacae*, in addition to a number of conjugative plasmids [20]. The *mrkABCDF* cassette has previously been identified on plasmids of the IncX1 group where the operon is likely to originally have been mobilized by a composite transposon, Tn*6011*, from the chromosome of *K*. *pneumoniae* onto an IncX1-type plasmid [21].

Here we examine the difference between type 3 fimbriae encoded on plasmids versus chromosomes, as little is known about the potential underlying differences. The understanding of biofilm-associated factors encoded on plasmids is currently inadequate as most research typically focuses on conserved biofilm-associated factors encoded by the chromosomes of various model bacteria. While such studies have generated important knowledge about the chromosomally encoded biofilm systems of a diverse array of bacteria, the chromosome of bacteria is normally complemented by a variety of extra-chromosomal elements such as plasmids, many of which transfer horizontally. Importantly, it has previously been shown that conjugative pili of incF plasmids enhance biofilm formation [22, 23], suggesting that promoting biofilm formation could be linked to the transfer success of plasmids. Here, we illustrate that type 3 fimbriae encoded on plasmids enhance the horizontal transfer rate by enforcing biofilm formation and thus cell-to-cell interactions, while leaving the host motile, further ensuring a high rate of encounters between donors and recipients. We suggest that this dual faceted phenotype thrives due to fundamental changes in the expression of the *mrkABCDF* operon of plasmids compared to that of chromosomes.

## Results and Discussion

### Most *E*. *coli* strains capable of excessive biofilm formation did so via plasmid encoded traits

A small strain library consisting of 75 veterinary *E*. *coli* isolates was screened for biofilm formation (CV assay). 7 of these isolates were found to be especially good at forming biofilms on the polystyrene surface of the microtiter plates. The threshold for “especially good biofilm formers” was set at an absorbance of OD_590_ > 1.0, which was approximately 10 times as much as *E*. *coli* MG1655 cells under the provided conditions. As the ability to form biofilm varied markedly among the veterinary strains, we hypothesized that the traits that enabled the hyper-biofilm forming behavior could be encoded on plasmids. Plasmids from the 7 biofilm-forming strains were therefore isolated and transformed into cells of *E*. *coli* GeneHogs^™^. 6 of the transformants showed a substantial increase in biofilm formation, suggesting that the biofilm-promoting traits of these 6 strains were encoded on plasmids. Performing gel-electrophoresis based on the plasmid DNA from the 6 strains indicated that only one plasmid was present in each strain. To avoid redundant sequencing, each of the plasmids was “fingerprinted” by restriction endonuclease digestion using *Eco*RI and *Nco*I followed by gel electrophoresis. Of the 6 plasmids analyzed, band patterns revealed that 3 of the 6 plasmids were unique. These three plasmids were hereafter fully sequenced using the 454 pyrosequencing platform and gaps between contigs were closed by Sanger sequencing. Two of the plasmids, pIS15_43 and pIS04_68, were found to encode type 3 fimbriae and are presented here. The 3^rd^ plasmid did not encode type 3 fimbriae, but other potential biofilm-promoting genes. Further characterization of this plasmid will be presented elsewhere.

### Nucleotide sequencing of plasmids pIS15_43 (IncX1) and pIS04_68 (IncR)

**Fig 1:**
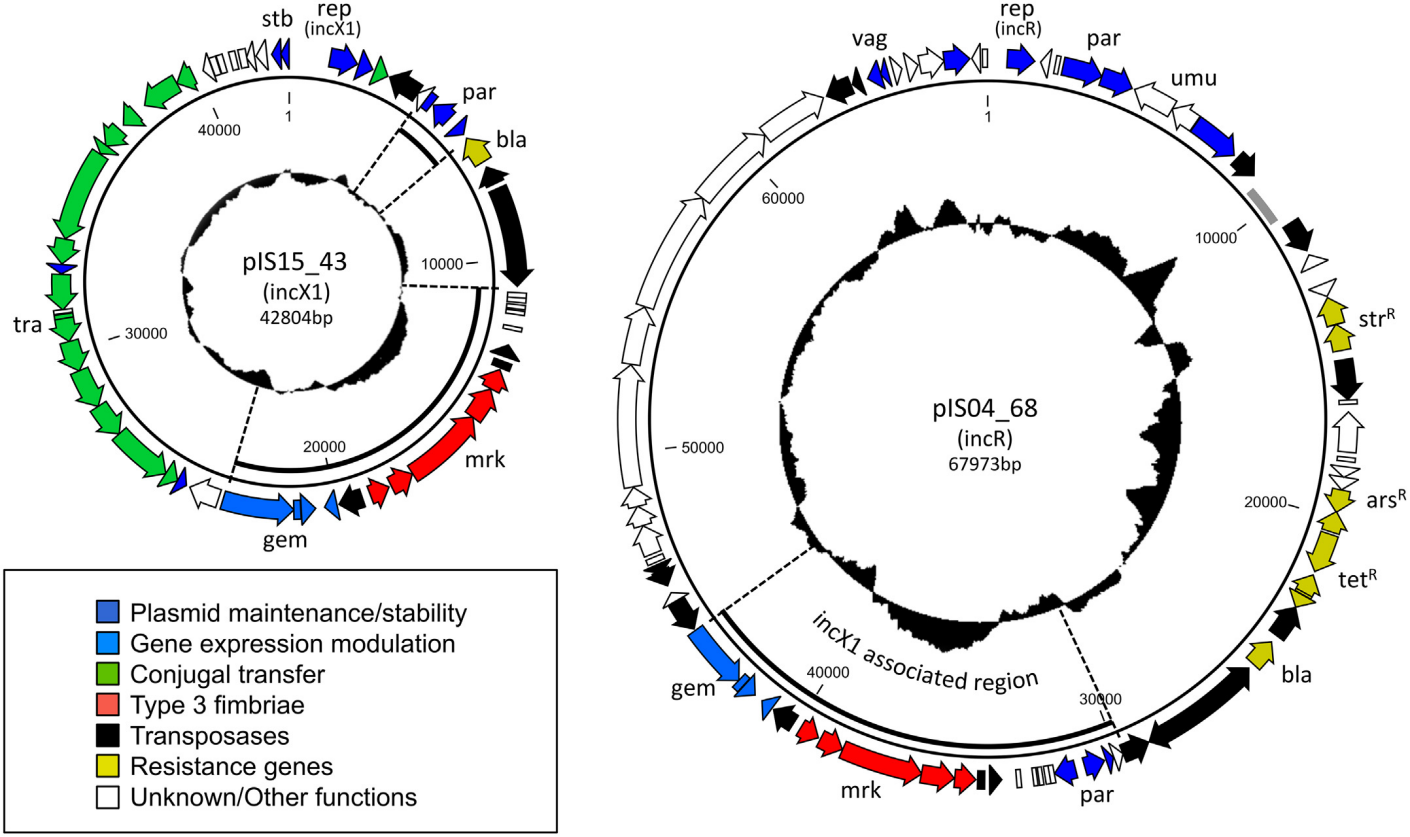
Graphic representation of annotated plasmids pIS15_43 and pIS04_68. %GC content is illustrated in the center of the plasmid diagrams. Regions of pIS04_68 (IncR) that are typically associated with the IncX1-type plasmids (such as pIS15_43) have been highlighted in both sequence maps.

Plasmid pIS15_43 (Fig 1) is a 42804 bp long conjugative plasmid showing high resemblance to other IncX1 plasmids, particularly pOLA52 (NC_024961), which has been thoroughly described [24, 25]. Annotation revealed 56 putative open reading frames (ORFs). Genetic load regions [26] included a beta-lactamase (*bla*) gene associated with a Tn*3* transposon and the type 3 fimbriae encoding *mrkABCDF* cassette flanked by IS (insertion sequence) elements.

Plasmid pIS04_68 (Fig 1) is a 67973 bp large circular plasmid with 76 putative ORFs. No conjugation or mobilization elements were identified on pIS04_68. pIS04_68 is a composite/truncated plasmid composed of 4 regions that each resemble other plasmids published in GenBank (i:pKP1780, ii:pYR1, iii:pOLA52 & iiii:p1658/97). (i) The bulk of backbone elements are associated with IncR plasmids (64.3–68 Kbp & 1–8.2 Kbp) including: the replication initiation protein (RepB), the stabilization toxin/antitoxin system (*vagCD*), the DNA breaking/joining resolvase (ResA), the partitioning locus (*parAB*), the DNA repair system (*umuCD*) and the reverse transcriptase (RetA). These backbone elements of pIS04_68 showed especially high nucleotide and structural resemblance with those of IncR plasmid pKP1780 [27] (DQ449578). (ii) A 14.4 Kbp large region (29.8–44.2 Kbp) encoded both backbone and load ORFs otherwise associated with IncX1 plasmids (Fig 1), which include: type 3 fimbriae (*mrkABCDF)*, a partitioning locus (*parFG*), a putative DNA invertase (*pIS04_68_35*), a putative degenerate EAL domain containing protein (*xeal*) and a gene cluster proposed to be involved in gene expression modulation (*gem*). This region includes putative genes encoding: DNA topoisomerase III (*topB*), a gene expression modulator (*hha*), and a transcriptional regulator (*h-ns*) [24]. (iii) One genetic load region (11.9–25.3 Kbp) was dominated by putative antibiotic resistance genes: streptomycin resistance (*strAB*), antibiotic biosynthesis monooxygenase (*ydjA*), tetracycline resistance (*tetABCD*) and beta-lactamase (*bla*). A similar region has been found on plasmid pYR1 [28]. (iiii) The 4^th^ region (46.6–62.9 Kbp) encodes conserved hypothetical ORFs with putative stability functions including: DNA repair ATPase (*pIS04_68_62*), endonuclease (*pIS04_68_63*), methyltransferase (*pIS04_68_64*) and Lon protease (*pIS04_68_66*). This region is highly similar to regions of p1658/97 [29].

### Unique *mrkA* promoter on plasmids enable high *mrkABCDF* expression independently of MrkH

To date, 9 plasmids that carry the *mrkABCDF* cassette have been fully sequenced and made available in GenBank. It has been described how the *mrkABCDF* cassette found on plasmids outside of *K*. *pneumoniae* is believed to have originated from a close relative of *K*. *pneumoniae*, where the *mrkABCDF* cassette was mobilized from its chromosome onto a plasmid via an IS composite transposon. This was mainly recognized due to a highly conserved positioning of IS elements upstream, and to a lesser degree downstream, of the *mrkABCDF* cassette [21]. The same consistency of IS element positioning flanking the *mrkABCDF* cassettes was found in both pIS15_43 and pIS04_68.

**Fig 2:**
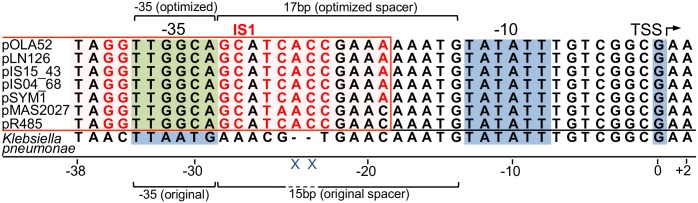
Nucleotide sequence alignment of *mrkA* promoters of plasmids from non-*Klebsiella* hosts (P_*mrkA*[P]_) and *K*. *pneumoniae* (P_*mrkA*[Kp]_). Residues that do not align with P_*mrkA*[Kp]_ are indicated in red, which are all associated with IS1 elements (red lining). Blue background highlights conserved P_*mrkA*[Kp]_ components and green background denotes the unique -35 box found in P_*mrkA*[P]_. TSS: transcriptional start site. P_*mrkA*[P]_ is predicted to be a stronger promoter than P_*mrkA*[Kp]_ due to an optimized -35 box and a 2 bp longer spacer region between the -10 box and -35 box.

Sequence analysis of all published *mrkABCDF* carrying plasmids revealed that the *mrkHI* and *mrkJ* genes were not present on any of these plasmids. Also, the MrkH box (GATCTATCAATG) and an AT-rich UP-element, both known to be important for MrkH mediated transcription induction [17], were not identified in any of the plasmids. Further analysis revealed conserved differences in the nucleotide sequence of the *mrkA* promoter region of the plasmids (P_*mrkA*[P]_) originating from non-*Klebsiella* hosts when compared to the *mrkA* promoter region of the *K*. *pneumoniae* chromosome (P_*mrkA*[Kp]_) (Fig 2). The distance from the start codon of *mrkA* to the transcriptional start site of the *mrkA* promoter is identical in both *K*. *pneumoniae* and plasmids (197 bp) and so is the -10 box of both P_*mrkA*[Kp]_ and P_*mrkA*[P]_. The -35 box of P_*mrkA*[P]_ is, however, different from P_*mrkA*[Kp]_. Comparative analysis suggests that the -35 box of P_*mrkA*[P]_ has been complemented with a different -35 box associated with the upstream IS1 element. Also the length of the spacer region between the -10 box and the -35 box is different in P_*mrkA*[P]_. When not induced by MrkH, Yang *et al*. [17] showed that P_*mrkA*[Kp]_ is a much stronger promoter if a 17 bp spacer was introduced instead of the 15 bp long spacer of the original promoter. The spacer of P_*mrkA*[P]_ was found to be 17 bp long (Fig 2) suggesting that changes to the spacer region of P_*mrkA*[P]_ in addition to the -35 box (Fig 2) could lead to changes in the promoter activity of P_*mrkA*[P]_ compared to P_*mrkA*[Kp]_.

**Fig 3:**
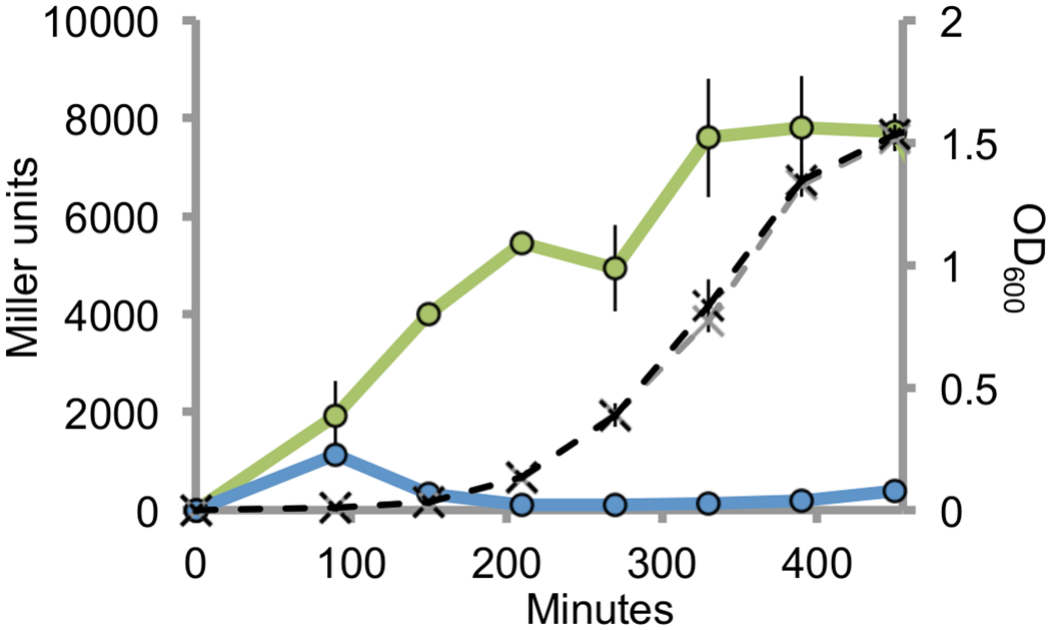
Transcriptional activity of promoters P_mrkA[Kp]_ and P_mrkA[P]_ in E. coli. β-galactosidase assays were performed after growth in LB at 37°C and samples were taken over a period of 8.5h. Growth (black) and β-galactosidase activity (green) of P_mrkA[P]_-lacZ fusion. Growth (gray) and β-galactosidase activity (blue) of P_mrkA[Kp]_-lacZ fusion (Error bars denote ±SEM, n = 3).

To study the properties of P_*mrkA*[P]_ and P_*mrkA*[Kp]_, each promoter was ligated in front of *lacZ* on plasmid pRS415 to create transcriptional fusions. The fusion constructs were hereafter transformed into *E*. *coli* GeneHogs^™^. P_*mrkA*[P]_ amplification was done using pOLA52 as the template and spanned the region from -55 to +151 relative to the transcriptional start site. *K*. *pneumoniae* C3091 was used as the template for P_*mrkA*[Kp]_. Expression profiles of each promoter fusion were assessed in LB medium at 37°C (Fig 3). The P_*mrkA*[Kp]_-*lacZ* fusion showed low β-galactosidase activity with a minor peak just before the onset of the exponential phase. Much higher β-galactosidase activities were observed with the P_*mrkA*[P]_-*lacZ* fusion, where the activity increased steadily until the end of the exponential phase, illustrating a strong expression profile. This indicates that the IS1 associated -35 box, in addition to the optimized 17 bp spacer between the -35 and -10 box, found on the plasmids, changed the efficiency of the promoter dramatically compared with the P_*mrkA*[Kp]_ promoter in hosts like *E*. *coli* where no MrkH is present [17]. We found, in agreement with previous studies, that P_*mrkA*[Kp]_ is a weak promoter when not induced by MrkH [17]. The P_*mrkA*[P]_ did, however, not require MrkH induction for efficient expression, suggesting that the *mrkABCDF* operon of the plasmids is not controlled and regulated in sync with other biofilm genes of the host.

### *mrkABCDF* plasmids from non-*Klebsiella* hosts do not encode *mrkHI* or *mrkJ*

Fig 3 shows that the *mrkABCDF* operons found on non-*Klebsiella* plasmids were expressed without induction by MrkH. We were therefore interested in understanding whether the *mrkABCDF* operon of plasmids in general is not associated with regulatory genes *mrkH*, *mrkI*, or *mrkJ*.

**Fig 4:**
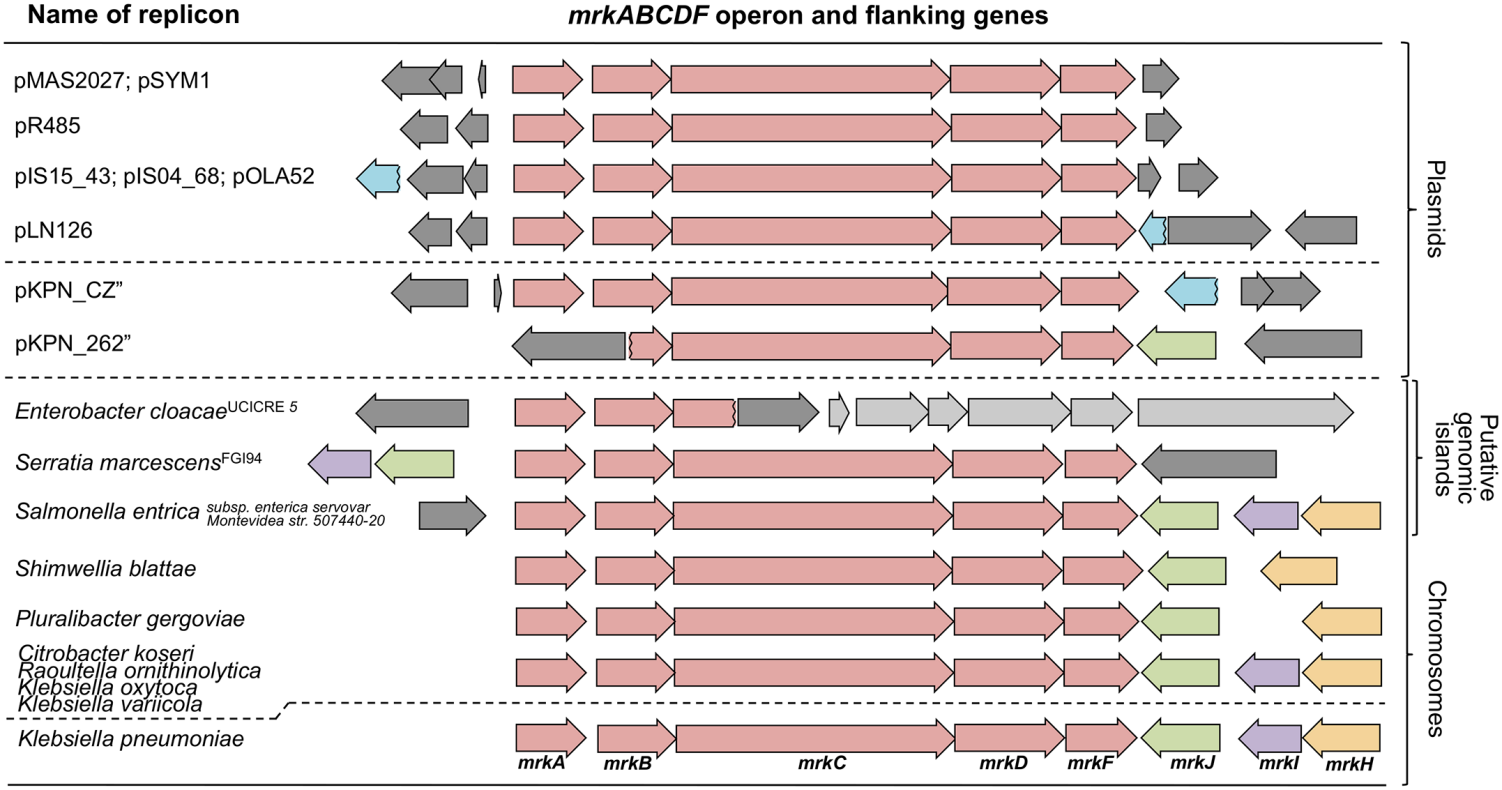
Genetic map of the *mrkABCDF* operon and flanking putative regulatory elements encoded on all fully sequenced representative replicons available in GenBank. Red: *mrkABCDF* genes. Orange: *mrkH* and *mrkH*_*L*_ genes. Purple: *mrkI* and *mrkI*_*L*_ genes. Green: *mrkJ* and *mrkJ*_*L*_ genes. Blue: putative genes encoding incomplete EAL domains. Dark gray: IS/transposon elements. Light gray: phage associated genes. (”) Plasmids pKPN_CZ and pKPN_262 have been isolated from *Klebsiella* hosts while all other plasmids originate from non-*Klebsiella* hosts.

Comparative analysis of available and completely sequenced replicons encoding the *mrkABCDF* operon revealed a large variety in the genetic architecture of putative *mrkH*-like (*mrkH*_*L*_), *mrkI*-like (*mrkI*_*L*_), and *mrkJ*-like (*mrkJ*_*L*_) genes flanking the *mrkABCDF* operon. Genes were classified as *mrkH*_*L*_ if the translated amino acid sequence holds a putative PilZ-type (cl01260) c-di-GMP binding motif and is classified as a putative transcriptional regulator. *mrkI*_*L*_ codes for putative proteins that have a LuxR motif (cl17315) and are transcriptional regulators. *mrkJ*_*L*_ genes encode putative proteins that hold an EAL-domain (cl00290). Fig 4 illustrates that 9 out of 10 chromosomally encoded *mrkABCDF* operons were flanked by putative *mrkH*_*L*_, *mrkI*_*L*_ and/or *mrkJ*_*L*_ genes, whereas only one *Klebsiella* plasmid (pKPN_262) encoded an intact *mrkJ*_*L*_ ORF. The type 3 fimbria operon of pKPN_262 is, however, not intact as *mrkA* and half of *mrkB* is missing likely due to disruption by a transposable element. The one exception among chromosomes was in the *Enterobacter cloacae* strain UCICRE 5 where only *mrkAB* and the beginning of *mrkC* were identified as part of a putative genomic island and flanked by IS elements and phage derived sequence, possibly transferred to this location by HGT. Fig 4 suggests that regulatory genes *mrkJ*, *mrkI* and *mrkH* are not selected for on plasmids in concert with *mrkABCDF*, possibly because these genes do not regulate P_*mrkA*[p]_ activity.

**Fig 5:**
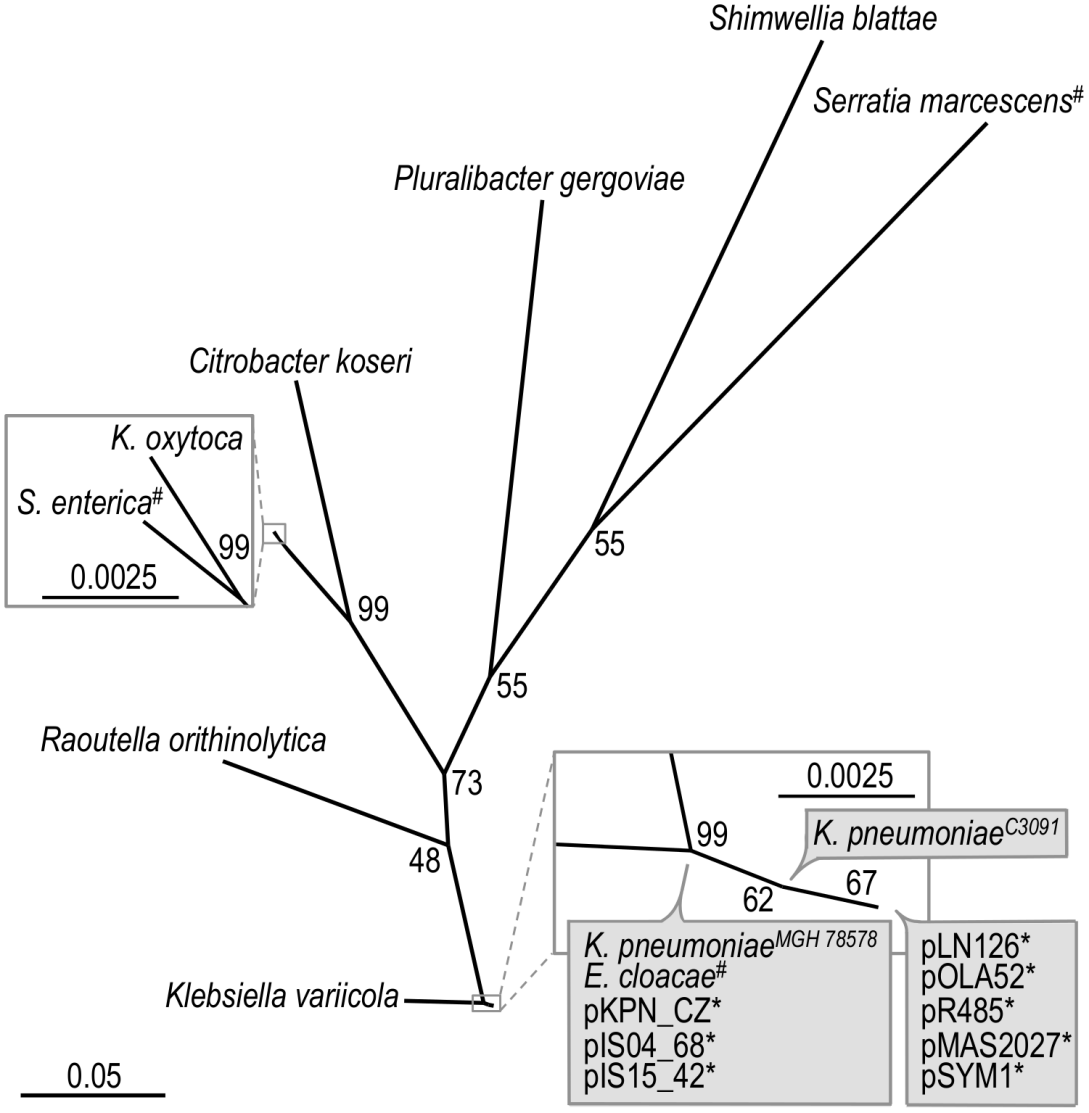
Maximum likelihood phylogeny based on the nucleotide sequence of PapD_N and PapD_C of *mrkB*. (*) Indicates that *mrkB* is encoded on a plasmid. (^#^) Indicates that *mrkB* may be part of a putative genomic island. Percentages of bootstrap support (1000 replicates) are shown.

Fig 5 is an unrooted phylogram tree based on the nucleotide sequence of the PapD_N (pfam00345) and PapD_C (pfam02753) domains of the *mrkB* genes. The tree is based on the same strains as were presented in Fig 4. This shows that the *mrkABCDF* operons found on plasmids are closely related to those of *K*. *pneumonia* and that the *mrkABCDF* operons encoded by chromosomes demonstrate higher diversity than those associated with plasmids, supporting the notion that the plasmid *mrkABCDF* operons have mobilized recently from a *K*. *pneumoniae-*like chromosome. Interestingly, *mrkB* of *E*. *cloacae* UCICRE 5 is grouped with both the plasmids and *K*. *pneumoniae*, suggesting that this could be a genomic island that might have been mobilized from a *K*. *pneumoniae-*like chromosome. The low diversity among the plasmid encoded *mrk* genes is possibly linked to the lack of regulatory activator *mrkH* on the plasmids and the strong MrkH independent expression observed from P_*mrkA*[p]_, suggesting a plasmid specific selection for the highly expressed P_*mrkA*[p]_-*mrkABCDF* cassette that does not need activation by MrkH.

### The P_mrkA[P]_*mrkABCDF* cassette enables abundant biofilm formation but does not influence motility

**Fig 6:**
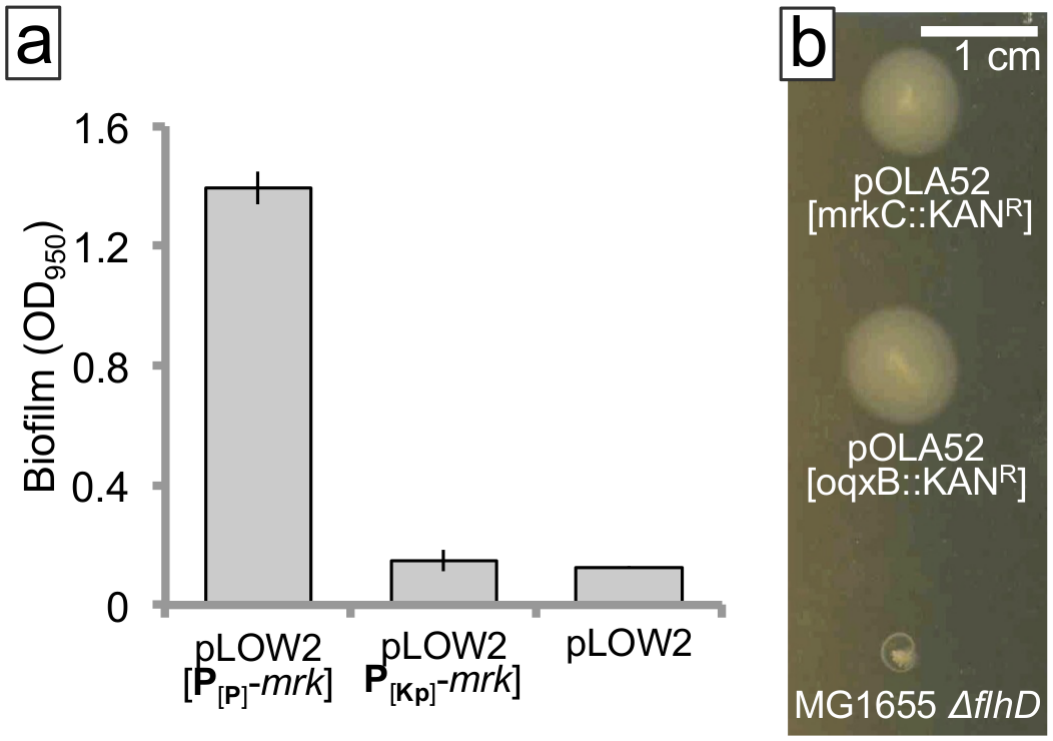
Influence of plasmid encoded *mrkABCDF* expression on biofilm-formation and motility of *E*. *coli*. (a) Biofilm formation by CV staining of attached cells after 24h growth at 37°C in microtitre plate wells. P_*mrkA*[P]_-*mrkABCDF* made more biofilm than both P_*mrkA*[Kp]_-*mrkABCDF* and the control: pLOW2 without *mrkABCDF*. (Error bars denote ±SEM, n = 3). (b) Swimming motility assay performed in 1% tryptone medium with 0.3% agar at 37°C. Negative controls (MG1655 *ΔflhD*) confirm the applicability of the assay.

**Fig 7:**
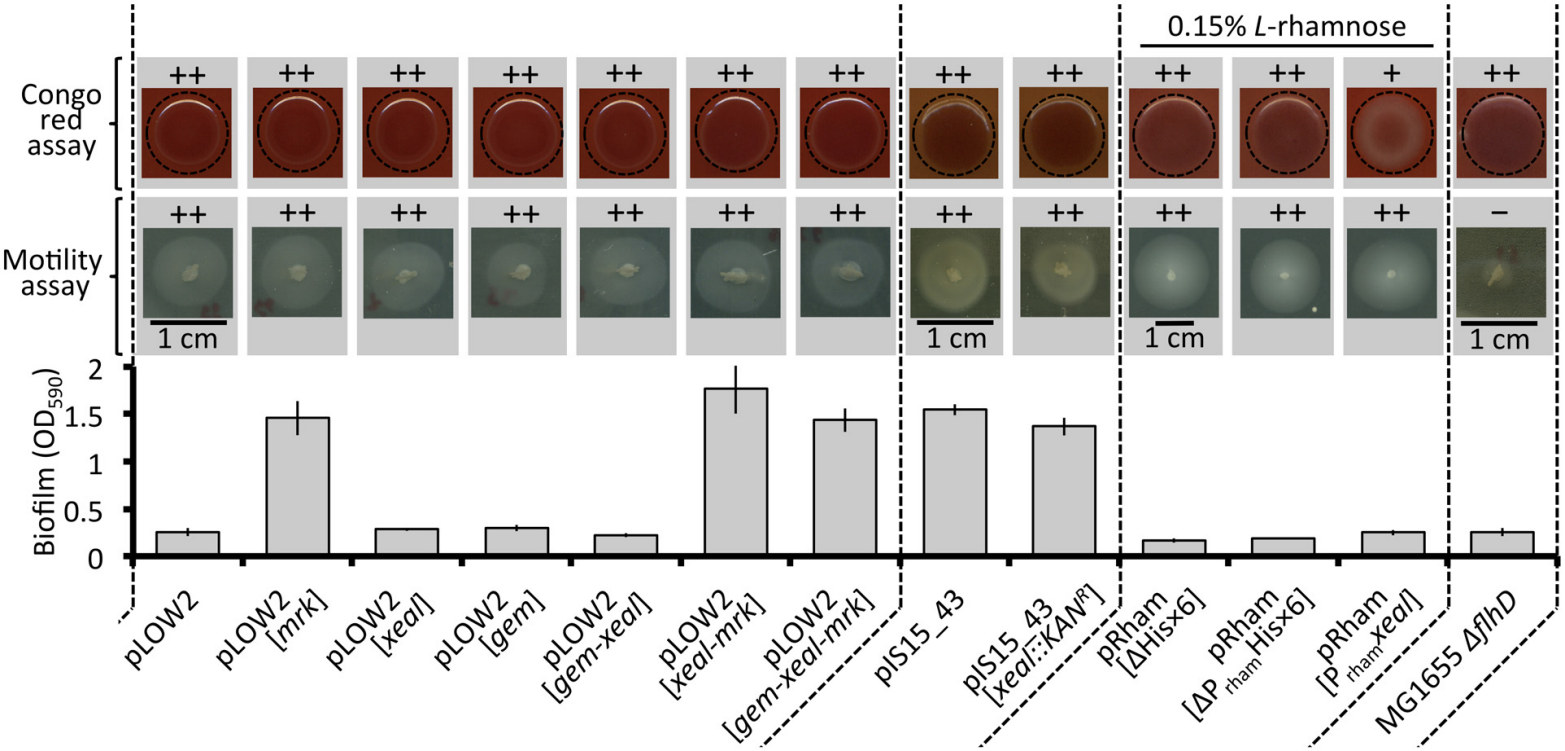
Examination of putative genes, all encoded on pOLA52, pIS15_43 and pIS04_68 that might influence the biofilm or motility behavior of the bacterial host. *Bottom*: CV biofilm assay. *E*. *coli* harboring constructs with the *mrkABCDF* cassette (*mrk*) formed more biofilm than those without but no difference was found among the biofilm formers (Error bars denote ±SEM, n = 3). *Middle*: swimming motility assay. Pictures were taken after 8h or 16h at 37°C. No differences were found (all swam equal distances, ++) with the exception of the negative control MG1655 *ΔflhD* (did not swim, −). MG1655 was used a positive control for swimming (not shown). *Top*: CR assay. Pictures were taken after 2 days at 30°C. No differences were found (equal amounts of CR was bound, ++) with the exception of pRham-P_*rham*_*xeal* for which less CR was bound by the colony (+). *Pseudomonas aeruginosa* PA14 and *P*. *aeruginosa* PA14 *Δpel* were used as positive and negative controls, respectively (not shown).

Motility and biofilm formation are opposing phenotypes, and bacteria such as *E*. *coli* either swim or form biofilm [3]. We, therefore, tested whether *E*. *coli* cells carrying an *mrkABCDF* encoding plasmid were also less motile, thus embracing a typical biofilm phenotype, or if the host would have an unusual phenotype, both expressing biofilm fimbriae and being actively motile. First, we tested if the promoter activities of P_*mrkA*[Kp]_ and P_*mrkA*[P]_ would directly correlate with low and high biofilm formation, respectively. Vector constructs based on the low copy number plasmid, pLOW2 [30], ligated with the P_*mrkA*[Kp]_-*mrkABCDF* or P_*mrkA*[P]_-*mrkABCDF* fragment were transformed into *E*. *coli* (GeneHogs^™^ and MG1655) and tested for biofilm formation in the crystal violet (CV) assay (Fig 6a). Biofilm formation was strongly induced only when *mrkABCDF* was expressed via the P_*mrkA*[P]_ promoter (see also Fig 7) as opposed to the P_*mrkA*[Kp]_-*mrkABCDF* construct, which made as little biofilm as the vector control (pLOW2 without *mrkABCDF* insertion). The swimming motility of the *E*. *coli* hosts was not influenced by the expression of the intact P_*mrkA*[P]_-*mrkABCDF* cassette of plasmid pOLA52 (pOLA52-*oqxB*::*KAN*^*R*^), compared with pOLA52 where *mrkC* has been disrupted by transposon insertion (pOLA52-*mrkC*::*KAN*^*R*^) (Fig 6b). We observed the same for cells of both *E*. *coli* GeneHogs^™^ and MG1655. Hence, no difference in swimming motility was found, illustrating that the P_*mrkA*[P]_-*mrkABCDF* cassette of wild type (WT) plasmids enforces an unusual phenotype of the hosts expressing both biofilm and planktonic phenotypic traits.

Putative genes that are found on multiple *mrkABCDF* plasmids were identified as candidates that can be linked to functions that might influence biofilm and/or planktonic behavior of *E*. *coli* (excluding the *mrkABCDF* cassette). These putative genes are part of the *gem* region (*hha* & *h-ns*) or *xeal* (Fig 1). Hha and H-NS have been shown to interact and hereby participate in the regulation of a number of genes influencing both biofilm formation and motility functions of *Enterobacteriaceae* [31–35]. *xeal* holds a putative degenerate c-di-GMP EAL domain and was therefore also investigated.

A number of vector constructs were created and tested in the CV, swimming motility and Congo red (CR) assays in order to assess if the aforementioned genes (*hha*, *h-ns* and *xeal*) affect host biofilm formation or motility. Congo red binds to curli fimbriae and cellulose, which are expressed from chromosome encoded genes during *E*. *coli* biofilm formation [36]. *E*. *coli* colonies therefore become red in the CR assay if curli fimbriae and/or cellulose is expressed. Fig 7 summarizes the results of these experiments. The overall conclusion is that none of the putative genes *xeal* or those of the *gem* region influenced the biofilm or planktonic motile behavior of the host under the tested conditions. The only deviation from this was that less CR was bound when *xeal* was highly expressed by an *L*-rhamnose inducible promoter. Although this is a potentially interesting observation, further analysis is required to clarify if this was an artifact of overexpressing Xeal, or if Xeal influences curli fimbriae and/or cellulose formation. Utilizing the other constructs that also hold *xeal*, no distinction was found between biofilm formation in the CV assay and the swimming motility assay. We therefore conclude that none of the putative genes that were annotated as potential biofilm or motility modulators (*hha*, *h-ns* and *xeal*) encoded on plasmids pOLA52, pIS15_43 and pIS04_68, influenced the performance of the hosts within the detection limits of the conditions provided here.

In order to corroborate the above findings we generated transcriptomes of *E*. *coli* MG1655 in exponential phase and compared transcription profiles in cultures with and without pIS15_43 (two biological replicates were made of each). Data is summarized in S3 and S4 Tables. Genes were considered differentially expressed if there was at least a 2-fold change in expression supported by P-value < 0.05.

First, the transcriptome data was used to verify that the P_*mrkA(p)*_ promoter prediction earlier (Fig 2) was correct (S1 Fig), which was done by mapping reads against the upstream region of *mrkA*. We found that transcription started around 194-204bp upstream of *mrkA*. The only likely P_*mrkA(p)*_ promoter for transcription to start this far upstream of *mrkA* is the one shown in Fig 2.

Overall, no consistent patterns of transcription indicated that biofilm or motility functions in general were up or down regulated differentially in plasmid bearing cultures compared to plasmid free ones, other than the plasmid encoded *mrkABCDF* operon. For example, the generated dataset suggests no significant differences in transcription for curli fimbriae (*csg*), colonic acid (*wza*/*cps*), cellulose (*bcs*), type 1 fimbriae (*fim*), flagellum (*flh*, *fli*, *flg*–except *flgB*), chemotaxic (*che*), GGDEF and/or EAL domain protein genes (*dos*, *rtn*, *yahA*, *yaiC*, *yedT*, *ycdT*, *ycgF*, *ycgG*, *yciR*, *ydaM*, *yddV*, *YdeH*, *ydiV*, *yeaI*, *yeaJ*, *yeaP*, *yedQ*, *yegE*, *yfeA*, *yfgF*, *yfiN*, *yhdA*, *yhjH*, *yhjK*, *yjcC*, *ylaB*, *yliE*, *yliF*, *yneF*, *yoaD*) or PilZ domain protein genes (*ycgR*, *bcsA*). Four non-conjoined genes that potentially are associated with biofilm and/or motility phenotypes were, however, found to be differentially expressed: *flu*, *flgB* and *hdfR* were less expressed in plasmid bearing cultures, 12.6, 2.4 and 2.7 fold less, respectively. *flu* encodes antigen 43, which has been shown to cause aggregation and inhibition of motility in *E*. *coli* [37]. *flgB* encodes a flagella component of the cell-proximal portion of the basal-body rod. Interestingly, *flgB* has been reported to be up-regulated in biofilms compared to cells in suspension [38]. *hdfR* encodes a H-NS dependent transcriptional regulator of the *flhDC* operon, which is the major regulator of flagella assembly and function [39]. However, the *flhDC* operon was not expressed differently in plasmid and plasmid free cultures. *plaP*, encoding a putrescine low affinity permease, was expressed 3.5 fold more in plasmid carrying cultures. Deletion of *plaP* has been shown to abolish swarming motility when all other predicted putrescine transporters were also deleted [40]. Given that the differential transcription of *flu*, *flgB*, *hdfR* and *plaP*, in theory, would lead to opposing biofilm/motility phenotypes makes it difficult to draw a consistent picture of how to interpret the significance of these observations. Taking together the lack of conjoined differential gene expression of biofilm or motility genes, revealed by the transcriptome data, with the phenotypes established in the previous sections (Fig 7), we deduct that the plasmid did not change the host behavior effectually besides enabling biofilm formation via plasmid encoded type 3 fimbriae.

### Conjugative plasmid transfer is enhanced by both motility and type 3 fimbriae

It seems that *mrkABCDF* encoding plasmids act by enforcing a change in key behaviors of their hosts while bypassing regulation. We therefore hypothesized that the unusual motile and fimbriae expressing phenotype is connected with the horizontal transmission success of the plasmid replicon. Transfer frequencies were compared between a conjugative plasmid with an intact *mrkABCDF* cassette (pOLA52-*oqxB*::*KAN*^*R*^) and a non-functional cassette (pOLA52-*mrkC*::*KAN*^*R*^). Both a motile (MG1655) and a non-motile (MG1655 *ΔflhD*) *E*. *coli* donor strain were used in order to imitate the more typical motile^+^/fimbriae^-^ (MG1655//pOLA52-*mrkC*::*KAN*^*R*^) and motile^-^/fimbriae^+^ (MG1655 *ΔflhD*//pOLA52-*oqxB*::*KAN*^*R*^) phenotypes, but also the unusual motile^+^/fimbriae^+^ (MG1655//pOLA52-*oqxB*::*KAN*^*R*^) phenotype inflicted by wild-type *mrkABCDF* plasmids in addition to the motile^-^/fimbriae^-^ (MG1655 *ΔflhD*//pOLA52-*mrkC*::*KAN*^*R*^) phenotype. *E*. *coli* MG1655 was used as recipient in all experiments.

**Fig 8:**
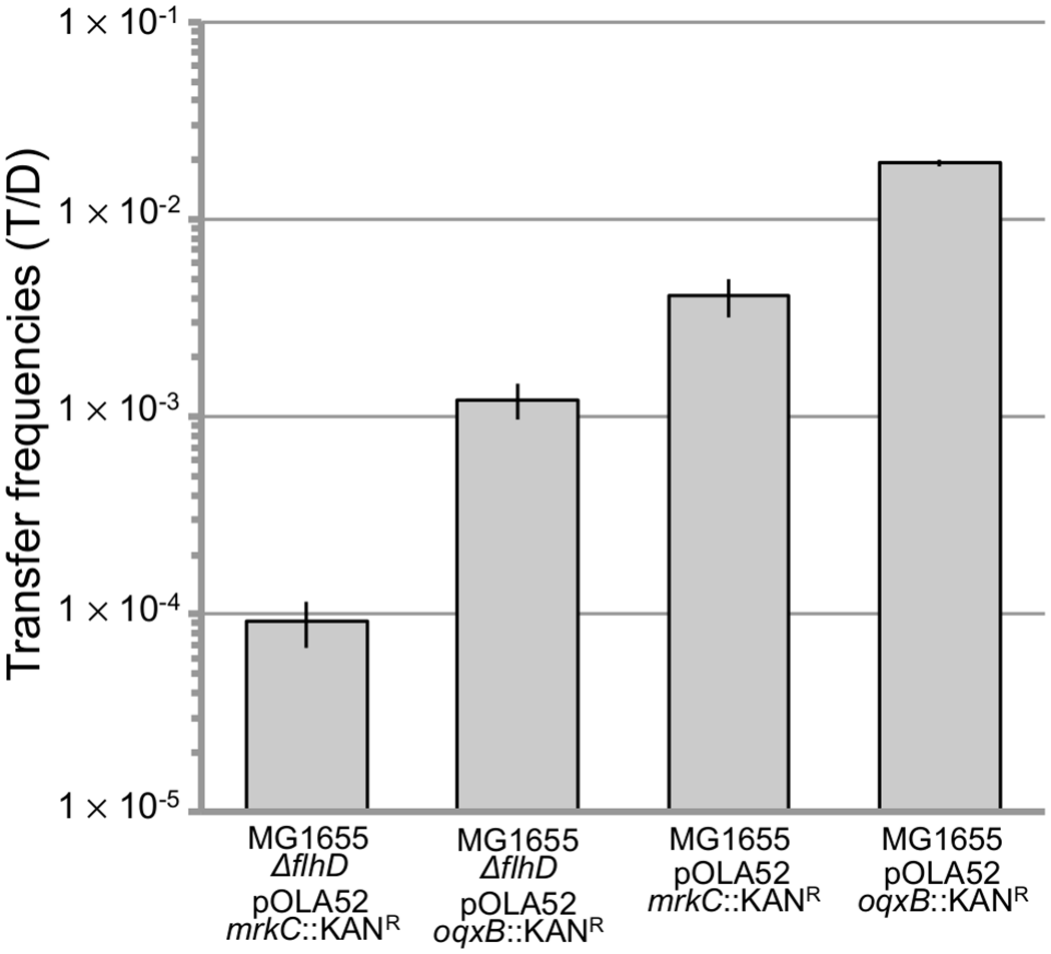
Conjugative transfer frequencies of pOLA52 with and without an intact *mrkABCDF* operon from motile and non-motile donors. Transfer frequencies were calculated as the number of transconjugants (T) per donor (D). *ΔflhD*: non-motile *E*. *coli* MG1655 *flhD* deletion mutant unable to synthesize flagella. MG1655: motile *E*. *coli* MG1655. pOLA52-*oqxB*::*KAN*^*R*^: type 3 fimbriae positive plasmid. pOLA52-*mrkC*::*KAN*^*R*^: type 3 fimbriae negative plasmid. Each transfer frequency was different from the other (Error bars denote ±SEM, n = 3).

The results are presented in Fig 8. Expression of the intact P_*mrkA[P]*_*mrkABCDF* cassette (pOLA52-*oqxB*::*KAN*^*R*^) enhanced the transfer rate of the plasmid compared to the *mrkC* knockout mutant strain (pOLA52-*mrkC*::*KAN*^*R*^) in accordance with previous findings [41]. Motility of the donor strain also had a large effect on conjugative plasmid transfer: pOLA52 was transferred at higher rates from the motile host than from the non-motile host. This was found for both the biofilm inducing plasmid (pOLA52-*oqxB*::*KAN*^*R*^) and the plasmid with a non-functional *mrkABCDF* operon (pOLA52-*mrkC*::*KAN*^*R*^). Intriguingly, the overall highest rates of transfer were observed by the conjugative pOLA52 with an intact *mrkABCDF* cassette and from the motile donor, which equates the wild-type *mrkABCDF* plasmids and a motile *Enterobacteriaceae* donor. Furthermore, the lowest transfer rates were found from the non-motile donor of the plasmid with the disrupted *mrkABCDF* cassette. These findings suggest that both biofilm characteristics and host motility enhance the probability of horizontal transfer of the plasmid.

In accordance, our transcriptome data shows that type 3 fimbriae (*mrk*), the conjugative machinery (*pilx*, *taxAB*) and flagella associated genes (*flh*, *fli*, *flg*) were all expressed during the late exponential phase in cells harboring the plasmid (S4 Table, average RPKMs: *mrk*: *337*, *pilx*: *18*, *taxAB*: *28*, *flh*: *17*, *fli*: *17* & *flg*: *16*). This supports the notion that the host acquires an exceptional phenotype, that both expresses biofilm and planktonic behaviors due to the acquisition of the *mrkABCDF* encoding plasmid, and that these phenotypes, transcription profiles, and elevated horizontal transfer rates of *mrk*-encoding plasmids are linked temporally.

### Concluding remarks

Transitioning between a planktonic and biofilm state is an example of phenotypic plasticity of bacteria. Phenotypic plasticity is typically directly connected to the fitness of organisms, as it enables a swift response to environmental change [42]. Mutation driven adaptation can lead to fixation of specific phenotypes and thereby reduce the phenotypic plasticity of bacteria [43]. Such fixation is known to occur when conditions in the local environment become more static, because selection then may favor a specific fixed phenotype and not plasticity [44].

Here we show that plasmid-encoded *mrkABCDF* enable bacteria to produce biofilm while retaining its ability to swim. Plasmid-encoded *mrkABCDF* genes are highly expressed without MrkH activation, which is a fundamental difference between *mrk-*operons on plasmids and chromosomes. *mrkABCDF* encoding plasmids, therefore, enforce a reduction in behavioral plasticity as the host is “locked” in a sticky biofilm-type behavior. This, however, is fundamentally different from mutations in the chromosome that induce the biofilm phenotype because the type 3 fimbriae are acquired by *E*. *coli* via HGT. Therefore, an entirely novel function is acquired by the *E*. *coli* host, which may increase the fitness of the host under specific selective pressures. Also, the phenotype that the plasmid imposes via the strongly expressed *mrkABCDF* operon was shown to enhance the horizontal transfer success of the plasmid and may thus be, at least in part, a plasmid selfish trait.

The long controversy of whether plasmids are molecular parasites or functional modules that only persists when complementing the host is therefore also in this case complex, as this is likely to primarily be determined by the level of local selective pressures and the degree of phenotypic plasticity that makes the host most fit. Plasmid encoded antibiotic resistance and toxin/antitoxin systems have been the focus of most research that examines the evolutionary dynamics of plasmid transfer, but these systems represent a very small fraction of the genes that are encoded on MGEs. Here we illustrate that factors such as fimbriae encoded by plasmids can also change the fundamental behavior of the host bacterium and argue that such factors are evolutionary successful not only because the host may become more fit in environments where adhesion is a selective advantage, but also because the horizontal transfer success of the plasmid is enhanced.

## Materials and Methods

### Bacterial strains and plasmids

The veterinary *Escherichia coli* strain-library used for the initial screen was kindly provided from the Danish integrated antimicrobial resistance monitoring and research program, DANMAP. 25 *E*. *coli* isolates were used from swine, cattle or poultry, respectively, and each isolate originated from a different animal (DANMAP 2008, [45]). All bacterial strains and plasmids are listed in S1 Table (supporting information). All bacteria used in this study were grown on Luria-Bertani (LB) agar (1.5%) medium at 37°C or in LB broth at 37°C at 250 rpm, unless otherwise stated. The following concentrations of antibiotics were used: Ampicillin (AMP); 100 μg/mL, Chloramphenicol (CHL); 50 μg/mL, Kanamycin (KAN); 50 μg/mL, Nalidixic acid (NAL); 100 μg/mL, Rifampicin (RIF); 100 μg/mL and Tetracycline (TET): 10 μg/mL.

### Crystal violet biofilm assay

Biofilm formation was quantified using a modified version of the Calgary assay previously described [46–48] using peg-lids (TSP) from NUNC. Incubation was done overnight at 37°C for 24h and staining was done with 1% crystal violet (CV). OD_590_ was used to measure CV absorbance on an EL 340 microplate reader (Bio-Tek Instruments).

### DNA manipulation techniques

Plasmid DNA was purified using the Plasmid Mini AX kit and genomic DNA was obtained using the Genomic Mini kit (A&A Biotechnology, Poland). PCR reactions were performed according to the standard protocol provided by Thermo scientific (Phusion Hot Start High-Fidelity DNA polymerase F540). Primers are shown in S2 Table. PCR fragments were extracted from agarose gels using QIAEX II Gel Extraction Kit (QIAGEN, Hilden, Germany). Enzymatic restrictions were performed according to standard procedures as stated by the manufacturer (New England Biolabs, Ipswich, USA). T4 DNA ligase was used for all ligation reactions following the provided protocol (New England Biolabs, Ipswich, USA). Electroporation with electro-competent *E*. *coli* Top10 and GeneHogs cells (Invitrogen) was done using a Gene Pulse apparatus (Bio-Rad). *E*. *coli* cells were made chemically competent and heat-shock transformed following standard protocols [49].

### Plasmid purification, sequencing and assembly of plasmids pIS04_68 and pIS15_45

Plasmid purification, sequencing and assembly was performed following the same steps as previously described [21]. *E*. *coli* GeneHogs transformants with either pIS04_68 or pIS15_43 were selected for on LB supplemented with AMP. pIS04_68 and pIS15_43 were sequenced on the GS sequencer FLX high throughput platform (454 Life Sciences, Branford, CT, USA). Single strand DNA libraries were constructed according to Roche protocols. Sequence assembly was performed with Newbler 2.6 (454 Life Sciences, CT, USA) and Consed [50]. Final assembly of contigs was done by Sanger sequencing (Macrogen, Korea) via PCR fragments that were made to span the gaps between the contigs. Primers are shown in S2 Table (supporting information). Accession numbers of pIS15_43 and pIS04_68 are NC_024961 and NC_024960, respectively.

### Annotations and sequence analysis

Annotation was done using Glimmer V3.02 [51] and CLC main workbench V6.9.1 (CLC Bio). General sequence analysis was done with the CLC main workbench software accompanied by Artemis [52], NCBI BLAST tools [53], BPROM (Softberry) and IslandViewer [54]. MEGA v.6.0,6 [55] was used to construct a Maximum likelihood phylogram, and Figtree v1.4.2 was used for visual representation. Accession numbers: CP007734, EU682505, NZ_KI535506, NC_013850, NC_018106, NC_021066, NC_009792, NC_020064, NC_017910, NC_010378, NC_019256, NC_019390, NC_013503, NC_016036, NC_019013, CP007530, and CP009450.

### Conjugative transfer experiments

Spontaneous RIF^R^ and NAL^R^ mutants were produced from the *E*. *coli* MG1655 [56] strain: 1 mL ON culture was spun down (5000 × *g*, 5 min), then re-suspended in 100 μL LB broth and spread on LB agar medium with RIF_100_ or NAL_100_. Single colonies were picked after ON incubation at 37°C and re-streaked multiple times to ensure pure clones. Purified plasmids pOLA52-*oqxB*::*KAN*^*R*^ and pOLA52-*mrkC*::*KAN*^*R*^ were transformed into the MG1655 RIF^R^ and MG1655 *ΔflhD* mutant strains. The four transformants were used as donors and the MG1655 NAL^R^ strain was used as the recipient in all conjugative transfer experiments. ON cultures of donor and recipient strains were adjusted to OD_600_ = 0.4, washed twice in LB broth, then adjusted to OD_600_ = 0.2 and left on ice for the duration of the mixing steps. Donor and recipient cultures were mixed to a ratio of 1:4 and incubated without any shaking at 37°C for 20h. Serial dilutions of the mating mixtures were hereafter prepared. Selective plating was done on LB agar medium using the following antibiotics: Transconjugants: NAL_100_ + KAN_50_. MG1655 RIF^R^ donor: RIF_100_ + KAN_50_. MG1655 *ΔflhD* donor: CHL_20_ + KAN_50_.

### Plasmid constructs and promoter fusions

Promoter fusions P_*mrkA*[Kp]_-*lacZ* and P_*mrkA*[P]_-*lacZ* were constructed by PCR amplifying the *mrkA* promoter region using *K*. *pneumoniae* C3091 and pOLA52, respectively, as DNA templates. All primers are shown in S2 Table. Promoter fusions were transformed into competent *E*. *coli* Top10 cells. Constructs pLOW2-P_*mrkA*[Kp]_*mrkABCDF*, pLOW2-*xeal*, pLOW2-*gem*, pLOW2-*xeal*-*gem*, pLOW2-*xeal*-P_*mrkA*[P]_*mrkABCDF* and pLOW2-*gem*-*xeal*-P_*mrkA*[P]_*mrkABCDF* were made using primers as specified in S2 Table. pOLA52 or pIS15_43 was used as a DNA template. pLOW2 based constructs were transformed into *E*. *coli* GeneHogs, Top10 and MG1655 strains. The Expresso^®^ Rhamnose Cloning and Expression System, C-His (Lucigen^®^, Wisconsin, USA) was used to construct pRham-P_*rham*_*xeal*, an *L*-rhamnose inducible *xeal* expression plasmid, following the guidelines provided by the manufacturer. Importantly, the *xeal* insert included a stop codon in order to avoid translation of the C-terminal His×6 tag region. pIS15_43 was used as a DNA template. Control plasmids, pRham-ΔHis×6 and pRham-ΔP_*rham*_His×6 were made by PCR amplifying the pRham vector, then digesting with *Bam*HI followed by ligation for re-circularization of the vector without the His×6 tag and the *L*-rhamnose inducible promoter plus His×6 tag, respectively. pIS15_43-*xeal*::*KAN*^*R*^ was constructed using the lambda red approach employing vector plasmids pUC4K [57] and pUCP-18-RedS [58].

### β-galactosidase activity assay

β-galactosidase activity was assayed as described elsewhere [59–61]. ON cultures of *E*. *coli* harboring pRS415-P_*mrkA*[Kp]_*lacZ* and pRS415-P_*mrkA*[P]_*lacZ* were diluted 1:1000 in LB-broth supplemented with AMP and grown at 37°C, 250 rpm to an OD_600_ of approximately 1.8. Samples were taken continuously over a period of 7.5h.

### Congo red assay

10 μL of ON culture was spotted on LB-agar plates without NaCl, with 0.5% NaCl and with 1.0% NaCl. The plates were supplemented with 40 μg/mL Congo red (CR)(Sigma) and 20 μg/mL Coomassie brilliant blue (Sigma). Incubation was done at 30°C for up to 4 days. *Pseudomonas aeruginosa* PA14 and *P*. *aeruginosa* PA14 *Δpel* were used as positive and negative controls, respectively.

### Swimming motility assay

The swimming motility assay was performed both in LB medium with 0.3% agar and in medium composed of 1% tryptone and 0.3% agar. 2 μL of ON culture was deposited into the agar-medium using a pipette tip. Plates were incubated at 37°C for 8h or 16h.

### RNA isolation, library construction, sequencing and data normalization

Total RNA was isolated from exponentially growing cultures (OD_600_ = 0.4) using RNeasy Mini Kit (Qiagen). No plasmid loss was observed in cultures with pIS15_43 although AMP_100_ was not added. This was tested by CFU counts on LB agar with and without AMP_100_ after the cultures had been in stationary phase for approximately 2h. (LB: 3.1×10^7^ ± 5.0×10^6^ CFUs, LB AMP_100_: 3.0×10^7^ ± 5.2×10^6^ CFUs, n = 3). Ribosomal RNA was reduced using the Ribo-Zero rRNA Removal Bacterial Kit (Epicentre Biotechnologies) according to the manufacturer’s protocol. The mRNA-enriched fraction was used as a template for preparation of indexed RNA-seq libraries using the ScriptSeq^™^ v2 RNA-Seq Library Preparation Kit (Epicentre Biotechnologies). The individual libraries were pooled and sequenced on a MiSeq platform using 2x75 PE v3 sequencing kit (Illumina). Obtained reads were trimmed and normalized with the CLC Genomic Workbench 7.5.1 (CLC). Briefly, reads were trimmed removing adapter sequences and discarding those of low quality using “trim sequences” tool (settings: ambiguous limit = 2, quality limit = 0.05). Afterwards, reads were depleted of rRNA sequences of the strain MG1655 (NC_000913). Next, un-mapped reads were mapped to the reference genome MG1655 and pIS15_43 plasmid (NC_024961) using the “map reads to reference tool” with the same settings as above. Finally, the genome-mapped and rRNA-depleted reads were normalized to the total number of 1.8 million reads for each replicate by random picking. RNA-seq analysis was performed in CLC Genomic Workbench 7.5.1 using “RNA-seq Analysis” tool with RPKM as the expression value. The differential expression experiment was set as two-group comparison with two replicates each (total nr of samples = 4). Group comparison of differentially expressed genes (DGE) was run on non-transformed data using a negative binominal model and exact testing as implemented in *edgeR* [62]. The false discovery rate (FDR) control was done according to Benjamini and Hochberg [63] and FDR-corrected p-values were considered for differentially expressed genes.

## Supporting Information

S1 Fig RNA-seq. reads mapped against the upstream region of *mrkA* (pIS15_43) verifying the predicted location of promoter P_*mrkA(p)*_. (DOCX)

S1 Table Strains and plasmids used in this study. (DOCX)

S2 Table Primers used in this study. (DOCX)

S3 Table Transcriptome data analysis of differential gene expression comparing plasmid harboring (pIS15_43) and plasmid free *E*. *coli* MG1655 cultures. (XLSX)

S4 Table Transcriptome data analysis of gene expression levels in plasmid harboring (pIS15_43) *E*. *coli* MG1655. (XLSX)
