# Supplementary material for: Type 3 Fimbriae Encoded on Plasmids Are Expressed from a Unique Promoter without Affecting Host Motility, Facilitating an Exceptional Phenotype That Enhances Conjugal Plasmid Transfer
Source: PLoS One. 2016 Sep 14;11(9):e0162390. doi: 10.1371/journal.pone.0162390 (PMC5023117; doi:10.1371/journal.pone.0162390)
Supplement: S1 Fig — (DOCX) [file pone.0162390.s001.docx]

**Supporting Information**

S1 Figure


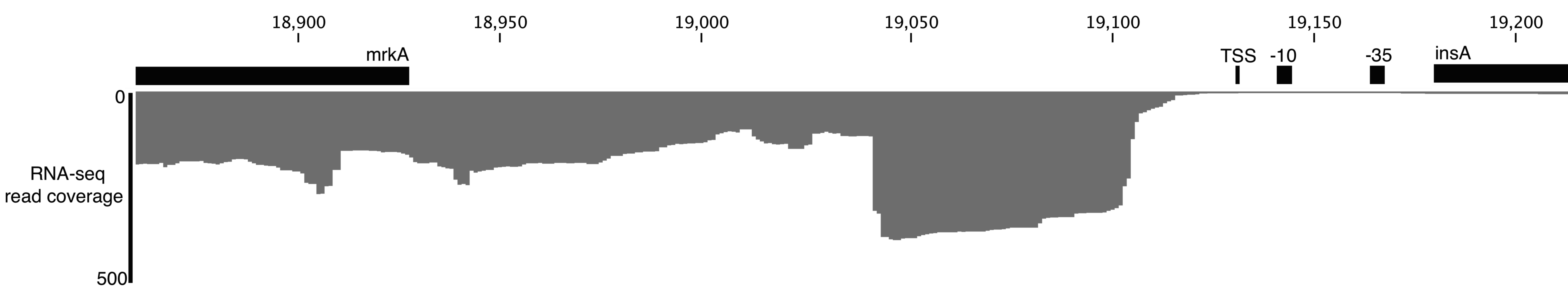


S1 Fig. RNA-seq. reads mapped against the upstream region of *mrkA* (pIS15_43) verifying the predicted location of promoter P*_mrkA(p)_*. See also Fig 2 for further details on P*_mrkA(p)_*_._
