# Supplementary material for: Type 3 Fimbriae Encoded on Plasmids Are Expressed from a Unique Promoter without Affecting Host Motility, Facilitating an Exceptional Phenotype That Enhances Conjugal Plasmid Transfer
Source: PLoS One. 2016 Sep 14;11(9):e0162390. doi: 10.1371/journal.pone.0162390 (PMC5023117; doi:10.1371/journal.pone.0162390)
Supplement: S1 Table — (DOCX) [file pone.0162390.s002.docx]

**Supplementary Information**

S1 Table - Strains and plasmids used in this study

| **Strain or plasmid** | **Characteristics/additional information** | **References** |
| --- | --- | --- |
| **Strains** |  |  |
| *Escherichia coli* IC01, IC02, …, IC25 | Isolate from the caecum of cattle in connection with slaughter | DANMAP 2008, ([1](#_ENREF_1)) |
| *Escherichia coli* IS01, IS02, …, IS25 | Isolate from the caecum of swine in connection with slaughter | DANMAP 2008, ([1](#_ENREF_1)) |
| *Escherichia coli* IP01, IP02, …, IP25 | Isolate from the caecum of poultry in connection with slaughter | DANMAP 2008, ([1](#_ENREF_1)) |
| *Escherichia coli* Genehogs | *F^–^ mcrA Δ(mcrCB-hsdRMS-mrr) φ80 lacZΔM15 ΔlacX74 deoR recA1 araD139 Δ(ara-leu)7697 galU galK rpsL (StrR) endA1 nupG fhuA::IS2* | Invitrogen |
| *Escherichia coli* Top10 | F*- mcrA Δ(mrr-hsdRMS-mcrBC) φ80lacZΔM15 ΔlacX74 nupG recA1 araD139 Δ(ara-leu)7697 galE15 galK16 rpsL(Str^R^) endA1 λ^-^* | Invitrogen |
| *Escherichia coli* MG1655, STR^R^ | *F^-^ λ^-^* *rph-1,* Spontaneous STR^R^ mutant of WT MG1655 | ([2-4](#_ENREF_2)) |
| *Escherichia coli* MG1655, STR^R^, RIF^R^ | Spontaneous RIF^R^ mutant of WT MG1655 STR^R^ | This study |
| *Escherichia coli* MG1655, STR^R^, NAL^R^ | Spontaneous NAL^R^ mutant of WT MG1655 STR^R^ | This study |
| *Escherichia coli* MG1655 Δ*flhD::cat*, STR^R^, CHL^R^ | Flagellum deficient mutant. *flhD* of WT MG1655 STR^R^ disrupted by insertion of *cat* (CHL^R^). This non-motile strain was used as negative control in swimming motility assays. | ([5](#_ENREF_5)) |
| *Escherichia coli* MG1655-ΔIS, STR^R^ NAL^R^ | IS*1* element associated with the *flhDC* promoter has been removed | ([6](#_ENREF_6)) |
| *Klebsiella pneumoniae* C3091, STR^R^ | Spontaneous STR^R^ mutant of UTI isolate, capsule serotype K16 | ([7](#_ENREF_7)) ([8](#_ENREF_8)) |
| *Pseudomonas aeruginosa* PA14 | Clinical isolate UCBPP-PA14, wild type. Binds high amounts of CR. Used as positive control in the CR assay | ([9](#_ENREF_9)) |
| *Pseudomonas aeruginosa* PA14 *Δpel* | ∆*phzA1-G1* ∆*phzA2-G2* deletion mutant. Does not binds CR. Used as negative control in the CR assay | ([10](#_ENREF_10)) |
| **Plasmids** |  |  |
| pIS15_43, AMP^R^ | Isolated from *E. coli* strain IS15 | This study |
| pIS04_68, AMP^R^, TET^R^, STR^R^ | Isolated from *E. coli* strain IS04 | This study |
| pOLA52, AMP^R^ |  | ([11](#_ENREF_11)) ([12](#_ENREF_12)) |
| pRS415, AMP^R^ | Multi-copy plasmid used for constructing *lacZ* fusions | ([13](#_ENREF_13)) |
| pRS415-P*_mrkA_*_[P]_*lacZ*, AMP^R^ | P*_mrkA_*_[P]_*lacZ* fusion. Plasmids: PmrkP_EcoRI_Fw & PmrkPK_BamHI_Rev. Endonucleases: *Eco*RI & *Bam*HI | This study |
| pRS415-P*_mrkA_*_[Kp]_*lacZ*, AMP^R^ | P*_mrkA_*_[Kp]_*lacZ* fusion. Plasmids: PmrkK_EcoRI_Fw & PmrkPK_BamHI_Rev. Endonucleases: *Eco*RI & *Bam*HI | This study |
| pOLA52-*oqxB*::KAN^R^, AMP^R^, KAN^R^ | Type 3 fimbriae positive, KAN^R^ entranceposon insertion in *oqxB* | ([14](#_ENREF_14)) |
| pOLA52-*mrkC*::KAN^R^, KAN^R^ | Type 3 fimbriae negative, KAN^R^ entranceposon insertion in *mrkC* | ([14](#_ENREF_14)) |
| pLOW2, KAN^R^ | Low-copy plasmid. Endonuclease based cloning vector | ([15](#_ENREF_15)) |
| pLOW2-P*_mrkA_*_[P]_*mrkABCDF*, KAN^R^ |  | ([14](#_ENREF_14)) |
| pLOW2-P*_mrkA_*_[Kp]_*mrkABCDF*, KAN^R^ | Primers used: K_mrkA_ApaLI_Fw & mrkRev. Endonucleases used: *Apa*LI & *Not*I. See also material and methods | This study |
| pLOW2-*xeal*, KAN^R^ | Primers used: Eal_mrk_Fw & xeal_notI_rev. Endonucleases used: *Apa*LI & *Not*I. See also material and methods | This study |
| pLOW2-*gem*, KAN^R^ | Primers used: topB_Fw & gem_rev . Endonucleases used: *Apa*LI & *Not*I | This study |
| pLOW2-*gem-xeal*, KAN^R^ | Primers used: topB_Fw & xeal_notI_rev. Endonucleases used: *Apa*LI & *Not*I. See also material and methods | This study |
| pLOW2-*xeal*-P*_mrkA_*_[P]_*mrkABCDF*, KAN^R^ | Primers used: Eal_mrk_Fw & mrkRev. Endonucleases used: *Apa*LI & *Not*I. See also material and methods | This study |
| pLOW2-*gem-xeal*-P*_mrkA_*_[P]_*mrkABCDF*, KAN^R^ | Primers used: topB_Fw & mrkRev. Endonucleases used: *Apa*LI & *Not*I. See also material and methods | This study |
| pRham, KAN^R^ | Plasmid with *L*-rhamnose inducible promoter. His×6 tag at the C-terminal. Homology based cloning vector. | Lucigen |
| pRham ΔHis×6, KAN^R^ | His×6 tag deleted and vector re-circularized. Primers: pRham_Rev-BamHI & pRham_FW_BamHI. Endonuclease: *Bam*HI | This study |
| pRham ΔP*_rham_*His×6, KAN^R^ | *L*-rhamnose inducible promoter plus His×6 tag deleted and vector re-circularized. Primers: pRham_Rev_BamHI_Pdel & pRham_FW_BamHI. Endonuclease: *Bam*HI | This study |
| pRham-*xeal*, KAN^R^ | *L*-rhamnose inducible *xeal* expression vector. Primers: Rha_xeal_FW & Rha_xeal_REV | This study |
| pUCP18-RedS, TET^R^ | Phage λ Red recombinase expression vector | ([16](#_ENREF_16)) ([17](#_ENREF_17)) |
| pKD4, KAN^R^ | Template plasmid for FLP-mediated excision of KAN^R^. Phage λ Red recombinase based cloning | ([16](#_ENREF_16)) ([17](#_ENREF_17)) |
| pIS15_43-*xeal*::KAN^R^, KAN^R^ | *xeal* disrupted by KAN^R^ insertion via phage λ Red recombinase based cloning. Primers used: EALNEXTF, EALNEXTR, k1, k2, & kt. See also material and methods | This study |

**References**

1. **Aarestrup FM, Bager F, Jensen NE, Madsen M, Meyling A, Wegener HC.** 1998. Resistance to antimicrobial agents used for animal therapy in pathogenic-, zoonotic- and indicator bacteria isolated from different food animals in Denmark: a baseline study for the Danish Integrated Antimicrobial Resistance Monitoring Programme (DANMAP). APMIS **106:**745-770.

2. **Miranda RL, Conway T, Leatham MP, Chang DE, Norris WE, Allen JH, Stevenson SJ, Laux DC, Cohen PS.** 2004. Glycolytic and gluconeogenic growth of *Escherichia coli* O157:H7 (EDL933) and *E. coli* K-12 (MG1655) in the mouse intestine. Infection and Immunity **72:**1666-1676.

3. **Guyer MS, Reed R, Steitz J, Low K.** 1981, p 135-140. Cold Spring Harbor symposia on quantitative biology.

4. **Leatham-Jensen MP, Frimodt-Møller J, Adediran J, Mokszycki ME, Banner ME, Caughron JE, Krogfelt KA, Conway T, Cohen PS.** 2012. The streptomycin-treated mouse intestine selects *Escherichia coli envZ* missense mutants that interact with dense and diverse intestinal microbiota. Infection and Immunity **80:**1716-1727.

5. **Leatham MP, Stevenson SJ, Gauger EJ, Krogfelt KA, Lins JJ, Haddock TL, Autieri SM, Conway T, Cohen PS.** 2005. Mouse intestine selects nonmotile *flhDC* mutants of *Escherichia coli* MG1655 with increased colonizing ability and better utilization of carbon sources. Infection and Immunity **73:**8039-8049.

6. **Gauger EJ, Leatham MP, Mercado-Lubo R, Laux DC, Conway T, Cohen PS.** 2007. Role of motility and the *flhDC* Operon in *Escherichia coli* MG1655 colonization of the mouse intestine. Infection and Immunity **75:**3315-3324.

7. **Oelschlaeger TA, Tall BD.** 1997. Invasion of cultured human epithelial cells by *Klebsiella pneumoniae* isolated from the urinary tract. Infection and Immunity **65:**2950-2958.

8. **Struve C, Bojer M, Krogfelt KA.** 2008. Characterization of *Klebsiella pneumoniae* type 1 fimbriae by detection of phase variation during colonization and infection and impact on virulence. Infection and Immunity **76:**4055-4065.

9. **Rahme L, Stevens E, Wolfort S, Shao J, Tompkins R, Ausubel F.** 1995. Common virulence factors for bacterial pathogenicity in plants and animals. Science **268:**1899-1902.

10. **Dietrich LEP, Price-Whelan A, Petersen A, Whiteley M, Newman DK.** 2006. The phenazine pyocyanin is a terminal signalling factor in the quorum sensing network of *Pseudomonas aeruginosa*. Molecular Microbiology **61:**1308-1321.

11. **Sørensen AH, Hansen LH, Johannesen E, Sørensen SJ.** 2003. Conjugative plasmid conferring resistance to olaquindox. Antimicrobial Agents and Chemotherapy **47:**798-799.

12. **Norman A, Hansen LH, She Q, Sørensen SJ.** 2008. Nucleotide sequence of pOLA52: a conjugative IncX1 plasmid from *Escherichia coli* which enables biofilm formation and multidrug efflux. Plasmid **60:**59-74.

13. **Simons RW, Houman F, Kleckner N.** 1987. Improved single and multicopy *lac*-based cloning vectors for protein and operon fusions. Gene **53:**85-96.

14. **Burmølle M, Bahl MI, Jensen LB, Sørensen SJ, Hansen LH.** 2008. Type 3 fimbriae, encoded by the conjugative plasmid pOLA52, enhance biofilm formation and transfer frequencies in *Enterobacteriaceae* strains. Microbiology **154:**187-195.

15. **Hansen LH, Sørensen SJ, Jensen LB.** 1997. Chromosomal insertion of the entire *Escherichia coli* lactose operon, into two strains of *Pseudomonas*, using a modified mini-*Tn*5 delivery system. Gene **186:**167-173.

16. **Datsenko KA, Wanner BL.** 2000. One-step inactivation of chromosomal genes in *Escherichia coli* K-12 using PCR products. Proceedings of the National Academy of Sciences **97:**6640-6645.

17. **Lesic B, Rahme L.** 2008. Use of the lambda Red recombinase system to rapidly generate mutants in *Pseudomonas aeruginosa*. BMC Molecular Biology **9:**20.
