# Supplementary material for: Type 3 Fimbriae Encoded on Plasmids Are Expressed from a Unique Promoter without Affecting Host Motility, Facilitating an Exceptional Phenotype That Enhances Conjugal Plasmid Transfer
Source: PLoS One. 2016 Sep 14;11(9):e0162390. doi: 10.1371/journal.pone.0162390 (PMC5023117; doi:10.1371/journal.pone.0162390)
Supplement: S2 Table — (DOCX) [file pone.0162390.s003.docx]

**Supplementary Information**

S2 Table - Primers used in this study

| **Primer name (restriction site)** | **Sequence 5’ 🡪 3’** | **Application** |
| --- | --- | --- |
| K_mrkA_ApaLI_Fw (*Apa*LI) | CGAGTGCACGGTCACTTTCTCTCGCT | pLOW2-P*_mrkA_*_[Kp]_*mrkABCDF* |
| PmrkP_EcoRI_Fw (*Eco*RI) | GACGAATTCCATACACTAAATCATTAGGTTG | pRS415-P*_mrkA_*_[P]_*lacZ* |
| PmrkK_EcoRI_Fw (*Eco*RI) | GACGAATTCGATGGTTATCTGTTATATAACTTA | pRS415-P*_mrkA_*_[Kp]_*lacZ* |
| PmrkPK_BamHI_Rev (*Bam*HI) | ATCGGATCCGCATAGAACCAGAAACAT | pRS415-P*_mrkA_*_[P]_*lacZ*  & pRS415-P*_mrkA_*_[Kp]_*lacZ* |
| Eal_mrk_Fw (*Apa*LI) | CGTGAAGTGCACCGTCCAATCCCACCCGAAAAACC | pLOW2-*xeal* & pLOW2-*xeal*-P*_mrkA_*_[P]_*mrkABCDF* |
| xeal_notI_rev (*Not*I) | GCAGCGGCCGCAGCGGCATAACCTGAATCTG | pLOW2-*xeal* |
| mrkRev (*Not*I) | GCAGCGGCCGCATTCCCGGCTTTTTAAC | pLOW2-P*_mrkA_*_[Kp]_*mrkABCDF*, pLOW2-*xeal*-P*_mrkA_*_[P]_*mrkABCDF* & pLOW2-*gem-xeal*-P*_mrkA_*_[P]_*mrkABCDF* |
| topB_Fw (*Apa*LI) | CGAGTGCACGATGCCCCACCTTTTTATTAGAG | pLOW2-*gem*, pLOW2-*gem-xeal* & pLOW2-*gem-xeal*-P*_mrkA_*_[P]_*mrkABCDF* |
| Gem_rev (*Not*I) | GCAGCGGCCGCATTCGGGTGGGATTGGACG | pLOW2-*gem* |
| Ealredf | GACAATGAAATTCAAAAAGTAATATCCAAAAATAAATTTATAGTGCTTGTGTAGGCTGGAGCTGCTTC | pIS15_43-*xeal*::KAN^R^ |
| Ealredr | TGTTCAGATGAAAACACATCTGCCTTCCGATATTTCATATATTTATAGCATATGAATATCCTCCTTAG | pIS15_43-*xeal*::KAN^R^ |
| EALNEXTF | GTGGAGCTGCATGACAAAGTCATC | pIS15_43-*xeal*::KAN^R^ |
| EALNEXTR | CGTAGCGCGCTGCCAGTAACTATC | pIS15_43-*xeal*::KAN^R^ |
| k1 | CAGTCATAGCCGAATAGCCT | pIS15_43-*xeal*::KAN^R^ |
| k2 | CGGTGCCCTGAATGAACTGC | pIS15_43-*xeal*::KAN^R^ |
| kt | CGGCCACAGTCGATGAATCC | pIS15_43-*xeal*::KAN^R^ |
| Rha_xeal_FW | GAAGGAGATATACATATGCTTGAAATATCAGAAACATAT | pRham-*xeal* |
| Rha_xeal_REV | GTGATGGTGGTGATGATGTTATAGCCACGCACTATTTAT | pRham-*xeal* |
| pRham_Rev-BamHI (*Bam*HI) | ACGGGATCCATGTATATCTCCTTCTTATAGTTAAA | pRham ΔHis×6 |
| pRham_Rev_BamHI_Pdel (*Bam*HI) | CAGGGATCCATCATACCTGACCTCCATAG | pRham ΔP*_rham_*His×6 |
| pRham_FW_BamHI (*Bam*HI) | TCAGGATCCCAATAACTAGCATAACCCCTT | pRham ΔHis×6 & pRham ΔP*_rham_*His×6 |
| pIS04 c160R | ATGCTGTAATGACTTGTGGT | pIS04_68 contig assembly |
| pIS04 c160F | CCCAACAATCTTTTACACGTC |  |
| pIS04 c282 | GGGGAGTCAGGCAACTATGG | pIS04_68 contig assembly |
| pIS04 c283F | CTTTTTTCTCAAATGCCACCG | pIS04_68 contig assembly |
| pIS04 c283R | AAGTAAAAAAGCAGAGAGTCCC | pIS04_68 contig assembly |
| pIS04 c283Rb | GCACAATACACAACCGTCTGA | pIS04_68 contig assembly |
| pIS04 c283Rc | CAGCCCGGTGGAATCAAT | pIS04_68 contig assembly |
| pIS04 c283Rd | CGCGAACAGCAATGAAGA | pIS04_68 contig assembly |
| pIS04 c283Re | GACACTCCGCCATACGACCA | pIS04_68 contig assembly |
| pIS04 c285F | ACCTGTTTTGCTGCACTTTCA | pIS04_68 contig assembly |
| pIS04 c285R | CATTAACCGACTTTGCGCGCT | pIS04_68 contig assembly |
| pIS04 c286F | TTTTCCAACGCCCAGTTTTC | pIS04_68 contig assembly |
| pIS04 c286R | GACGAAAGCAAACAAACTGG | pIS04_68 contig assembly |
| pIS04 c286Rb | AGCAAAATCCCGTCTGACATA | pIS04_68 contig assembly |
| pIS15 c001F | TGGGCAACGTGGGAATTA | pIS04_68 & pIS15_43 contig assembly |
| pIS15 c001R | GGCGTCAGGTAACGAACCTA | pIS04_68 & pIS15_43 contig assembly |
| pIS15 c002F | GTAAGAGAGCAAAGAAATGGT | pIS04_68 & pIS15_43 contig assembly |
| pIS15 c002R | AGAAGTAGTAGGCAGAGCAG | pIS04_68 & pIS15_43 contig assembly |
